# Identification of histological threshold concepts in health sciences curricula: Students' perception

**DOI:** 10.1002/ase.2171

**Published:** 2022-02-08

**Authors:** Miguel A. Martin‐Piedra, Salvador Saavedra‐Casado, Antonio Santisteban‐Espejo, Fernando Campos, Jesus Chato‐Astrain, Oscar Dario Garcia‐Garcia, David Sanchez‐Porras, Juan de Dios Luna del Castillo, Ismael Angel Rodriguez, Antonio Campos

**Affiliations:** ^1^ 16741 Tissue Engineering Group Department of Histology Faculty of Medicine University of Granada Granada Spain; ^2^ Instituto de Investigación Biosanitaria, ibs.GRANADA Granada Spain; ^3^ 16741 Doctoral (Ph.D.) Program in Biomedicine Faculty of Medicine University of Granada Granada Spain; ^4^ Department of Pathology Puerta del Mar University Hospital Cádiz Spain; ^5^ Institute of Research and Innovation in Biomedical Sciences of the Province of Cadiz (INiBICA) University of Cádiz Cádiz Spain; ^6^ 16741 Department of Biostatistics Faculty of Medicine University of Granada Granada Spain; ^7^ Department of Histology B Faculty of Dentistry National University of Cordoba Cordoba Argentina

**Keywords:** health sciences curricula, histology, histology education, medical education, students' perception, teaching and learning, threshold concepts, undergraduate education

## Abstract

Students' metacognitive skills and perceptions are considered important variables for high‐quality learning. In this study, students' perceptions were used to identify histological threshold concepts (integrative, irreversible, transformative, and troublesome) in three health sciences curricula. A specific questionnaire was developed and validated to characterize students' perceptions of histological threshold concepts. A sample of 410 undergraduate students enrolled in the dentistry, medicine, and pharmacy degree programs participated in the study. Concepts assessed in the study were clustered to ten categories (factors) by exploratory and confirmatory factor analysis. Concepts linked to tissue organization and tissue functional states received the highest scores from students in all degree programs, suggesting that the process of learning histology requires the integration of both static concepts related to the constituent elements of tissues and dynamic concepts such as stem cells as a tissue renewal substrate, or the euplasic, proplasic and retroplasic states of tissues. The complexity of integrating static and dynamic concepts may pose a challenging barrier to the comprehension of histology. In addition, several differences were detected among the students in different degree programs. Dentistry students more often perceived morphostructural concepts as threshold concepts, whereas medical students highlighted concepts related to two‐dimensional microscopic identification. Lastly, pharmacy students identified concepts related to tissue general activity as critical for the comprehension and learning of histology. The identification of threshold concepts through students' perceptions is potentially useful to improve the teaching and learning process in health sciences curricula.

## INTRODUCTION

The identification of students' perceptions constitutes a crucial element for the appropriate design and implementation of pedagogical strategies. Given that the learning process depends on triggers such as motivation (Wouters et al., 2016), self‐recognition of progress (Berkovich‐Ohana et al., 2019), and awareness of professionalism (Neve et al., 2017), the assessment of students' perceptions is an essential aspect of this process. The role of the student in the learning process has been examined from many different points of view in recent years (Campos et al., 2018; Agarwal & Kaushik, 2020; Rafati et al., 2020; Sun et al., 2020). Metacognition, defined as self‐knowledge of how the mind works and the intentional control of cognitive processes, has been identified as a relevant component for students' learning (Bryce et al., 2015; Vettori et al., 2018). Motivation and conceptions of learning are considered self‐regulatory constructs that arise from metacognition and that may closely influence student's learning, and consequently their academic outcomes (Martin & Ramsden, 1987; Vettori et al., 2018). Students' strategies aimed at seeking meaning and relating ideas (reconstructive conception) tend to achieve higher‐quality learning outcomes compared to students who practice unrelated memorizing (reproductive conception) (Entwistle et al., 2000; Zeegers, 2004). Accordingly, students' metacognitive skills and perceptions are considered important variables for high‐quality learning (Efklides, 2011; Campos‐Sanchez et al., 2012, 2014a, 2014b; Al Khader et al., 2020).

One of the learning theories that situates the student as the central element in learning is Threshold Concepts (TC) theory (Meyer & Land, 2003), which has been a topic of increasing interest for the scientific community in the last decade (Santisteban‐Espejo et al., 2020). Threshold Concept theory considers education as a space of uncertainty, and proposes the existence of certain concepts or learning experiences that resemble conceptual gateways or portals that lead to a previously inaccessible way of thinking about something (Meyer & Land, 2003, 2005). A specific notion can be considered a TC if it is transformative: once understood, these concepts trigger a significant shift in the student's perception of a subject, as well as emotional and performative elements in the learning process (Mezirow, 1990). In addition, TC are irreversible: once learned, they are unlikely to be forgotten by students. They are integrative, which means that their acquisition usually discloses previously hidden interrelations between apparently distant subjects. Lastly, TC are troublesome for learners because their learning is perceived as difficult or dissonant (Meyer & Land, 2003). The identification of specific TC within a discipline may thus be viewed as a relevant tool for focused curricular redesign, given that the teaching of these concepts can significantly improve students' learning (Entwistle, 2008). However, the identification of TC in health sciences currently constitutes a major challenge for higher education, because of the lack of a standardized, validated method for this purpose (Santisteban‐Espejo et al., 2020). Although TC have initially been identified by professors and staff scholars (Davies & Mangan, 2007; McKillop et al., 2014) students' perception may also play a key role in their identification, and students' perceptions are now increasingly used in the scientific community to identify TC (Clouder, 2005; Loertscher et al., 2014; Park, 2014). In this connection, several authors have attempted to identify TC by investigating the perceptions of medical students after conducting clinical practice in palliative care (O’Callaghan et al., 2020) and pediatrics (Randall et al., 2018). Personal reflections using audio diaries and subsequent group discussion have also been used to identify TC in bioethics (Collett et al., 2017), and to understand students' experiences in medical professionalism (Neve et al., 2017). Nevertheless, the question of how to appropriately identify TC still remains unresolved (Barradell, 2013; Santisteban‐Espejo et al., 2020). Attempts have been made to identify TC in clinical subjects as a pedagogical tool that may improve teaching in health sciences curricula; however, there is a lack of evidence regarding the identification of TC in the field of teaching histology.

In biomedicine, histology is a basic science that deals with concepts and facts regarding the microscopic structure of the human body. An understanding of histology is crucial to comprehend human biochemical and physiological processes, as well as to gain insights into how structural abnormalities lead to disorders resulting in disease (Shaw & Friedman, 2012; Lowe et al., 2019; Al Khader et al., 2020). As a basic science, histology constitutes a crossroad among other curricular contents in basic and clinical sciences. Accordingly, histology is a fundamental part of most syllabi in biomedical disciplines and can be considered a core cognitive element in health sciences curricula (Moxham et al., 2018; Cui & Moxham, 2021). In the health sciences curricula, histology offers the student the possibility of approaching and understanding the microscopic structure and ultrastructure of normal human cells and tissues as the basis of human pathology. In general, the study of the four basic tissues of the human body, i.e., the epithelial, connective, muscle and nerve tissues, is considered in all histology curricula. However, there is no international consensus on the exact contents that should be included in a histological curriculum. In this regard, a recent study by Cui and Moxam using Delphi panels was able to determine the most relevant matters of medical histology, and authors were able to propose a core syllabus for this discipline (Cui & Moxham, 2021). As expected, the study of human cells and basic tissues was considered within this syllabus, but several specialized tissues corresponding to the skeletal muscle, cartilage, bone, blood, bone marrow and other structures of the human body were also considered with 100% of consensus of the Delphi panel.

In addition, several strategies have been reported for the optimization of teaching in histology, such as the use of virtual microscopy (Mione et al., 2013), electronic learning resources (Ali & Syed, 2020) and the correlation with clinical cases (Eurell et al., 1999).

The teaching of histology has been enriched by interesting approaches such as functional and dynamic histology (Vandevelde, 2015; Varga et al., 2016). These pedagogical strategies consider that the teaching and learning process in histology should not be oriented in a traditional way that requires students to reproduce the components of a tissue in an unchanging or fixed mode, but rather should facilitate comprehension of the physiological and clinical significance of different tissue structures (Kerr, 2010). Consequently, the design and implementation of histology teaching programs could be significantly optimized by taking into account this dual, static–dynamic dimension of the subject matter.

Because of the many interdisciplinary relationships involved in this subject, learning histology could be significantly improved by the identification of TC. Ideally, these concepts should focus directly on the most integrative and troublesome aspects for students in order to optimize transformative, irreversible learning about microscopic structures in the human body. Given that improvements in educational programs are grounded on an accurate analysis of students' understanding of the subject matter, the evaluation of students' perceptions might constitute an appropriate tool to identify histological TC. Once identified, these concepts can then be used to construct the foundations for the further comprehension of clinical and surgical subjects. The results of the present study may have the potential to impact both basic and clinical sciences in higher medical education, given that knowledge of histology is essential for further progress in learning basic curricular contents intended to provide an understanding of the architecture of damaged tissues in different human diseases.

Histological concepts are also closely related to magnifying instruments (microscopes) and histological techniques (staining). Thus, this instrumental and methodological dimension is conjoined to histology, and should not be overlooked in the identification of histological TC.

However, the interrelations among histology and other disciplines are necessarily different in health sciences curricula, in which the course contents and goals are specific for each degree. In fact, guidance on how histology is taught differs across curricula, and focuses on different aspects within each degree program. For this reason, it is important to identify TC not only for each subject, but also for each academic degree program that includes a give subject.

In the present study TC were investigated during the teaching and learning process—an approach that made it possible to evaluate students' effort and cognitive integration as they occurred. Students were asked to identify histological TC after the histology course had been taught in its entirety. The undergraduate histology course in the health sciences curricula at the University of Granada ranges from 60 hours in the medicine and dentistry degree programs to 30 hours for the pharmacy degree (combined with 30 hours of anatomy in a unique 60‐h course). Part of the histology course consists of 10 to 15 hours of work in practical sessions to learn how to identify basic human tissues and their main characteristics in microscope slides, with both light microscopes and virtual microscope tools. Content knowledge is evaluated with theoretical and practical tests at the end of the course.

The present work aimed to evaluate students' perceptions regarding a group of concepts that are considered elemental in histology in different health sciences curricula, i.e., dentistry, medicine and pharmacy. Insights into students' perceptions should be useful in efforts to redesign and optimize the pedagogical methods and approaches used in the teaching and learning of histology.

## MATERIALS AND METHODS

### Design of the study

The present research was carried out to identify perceptions of TC in histology by students enrolled in undergraduate degree programs in medicine, dentistry and pharmacy. The study took place during the period when the histology course was taught; that is, the same module of histological concepts was taught to all students by the same teaching faculty members in the same period of the academic year. All students received the same information about the goals of the study and the procedure to be used. This study complied with the Helsinki Declaration and was approved by the Ethics Committee on Human Research of the University of Granada (ref. n. 622/CEIH/2018).

### Participants in the study

The study was done at the University of Granada (Spain). The sample consisted of 410 undergraduate students enrolled in the first year of three health sciences degree programs. Because general histology at the University of Granada is only taught as a full course in the first year of the degree program, only first‐year students were enrolled in this study. A total of 244 medical students (97.21% of all first‐year students), 64 dentistry students (94.11%), and 102 pharmacy students (88.70%) participated. The medicine, dentistry and pharmacy degree programs were chosen for this study because they share a similar syllabus for health sciences curricula, both at the University of Granada and at other universities in Spain. Mean age of the participants ranged from 18.7 ± 1.7 years in pharmacy to 19.5 ± 4.3 years in dentistry, with no significant differences among degree programs. The male/female ratio was similar in all three groups of participants (*P* = 0.456). Further information on the participants' demographic and university access characteristics are shown in Table 1. All participants received information about the definition of TC before they completed the questionnaire, which was distributed to students at the end of the academic year once all theoretical contents in the histology course had been taught. Participation was voluntary and consistent with the procedures of the university research review board. The students were given no extra credit or compensation for participating, and they were informed that their participation would help them explore their own learning processes.

**TABLE 1:** Demographic and higher education profile

Variable	Dentistry	Medicine	Pharmacy	*p*‐values
Number of participants	64	244	102	
Age in years Mean (±SD)	19.5 (±4.3)	19.3 (±1.8)	18.7 (±1.7)	0.063
Sex				0.456
Male *n* (%)	17 (26.6)	83 (34.0)	36 (35.3)	
Female *n* (%)	47 (73.4)	161 (66.0)	66 (64.7)	
Higher education access				
Regular *n* (%)	50 (78.1)	229 (93.9)	102 (100)	
Qualification Mean (±SD)	12.34 (±0.44)	12.97 (±0.35)	11.21 (±1.58)	<0.001

Note

Demographic and higher education profile of the *n* = 410 students enrolled in undergraduate degree programs in dentistry, medicine and pharmacy who participated in this study is shown. Regular Access mean the access to University from secondary education. Qualification for regular access is a score, ranged from 0 to 14 points, depending on the results of applicants in previous courses and the results of the University Entrance Examinations.

### Questionnaire tool

To identify histology TC, a tool named the Histological Threshold Concepts questionnaire (HTCq) was developed by faculty members of the Department of Histology at the University of Granada. (The questionnaire is available for use as long as this article is cited). This survey is registered in the Safe Creative Electronic Register of Intellectual Property (Saragossa, Spain) on November 5, 2021, code 2111059726532. This questionnaire consists of 37 concepts previously used in histology teaching activities. Students were asked to indicate the value of each concept as a TC on a five‐point Likert scale from 1 (total disagreement) to 5 (total agreement). Definitions were provided below each concept in the questionnaire. The HTCq (Figure S1) was completed after the last class of the histology course. The students were first informed of the aim of the HTCq and given instructions about how to complete it.

Although the HTCq presented students with 37 individual concepts, the constitution of groups of concepts (i.e., factors) was used to analyze the responses. These factors were obtained through statistical analysis, namely main components factor analysis.

Main components analysis was used to identify subgroups of items (factors) that were highly correlated. The aim was to obtain the minimum number of factors able to explain the greatest proportion of variance in the original set of items. This method consecutively identified factors which explained decreasing proportions of the overall variance (i.e., at the end of the process the number of factors is the same as the number of items), and assigned a quality score value to each factor (called the eigenvalue). Only factors with a high eigenvalue (i.e., greater than 1, in accordance with the Kaiser rule) were considered informative, and therefore retained. This procedure identified 10 factors that were retained. To identify which items pertained to each of the 10 factors, the correlation coefficients between each item and each factor were obtained. Each item produced a value for this coefficient (called the factor load) for each of the 10 factors retained. Ideally, each item should yield a high load on only one factor and very low loads on the remaining factors, although sometimes the same item showed high loads (i.e., greater than 0.40) on two or even more factors. To minimize this situation (which makes it difficult to distribute the items across the extracted factors, and consequently makes their interpretation challenging) and favor the optimal distribution of loads, the mathematical procedure called varimax rotation was first applied.

The model obtained from the results was contrasted with different confirmatory factor models derived from the different variants to be tested. The goodness of fit index (GIF), adjusted goodness of fit index (AGIF), and root mean square error of approximation (RMSEA) coefficient were used to verify the fitness of each proposed model, and thus to validate the underlying factorial structure of the HTCq tool (Bollen, 1989). These indexes and coefficients provide a measurement of fitness of the results model yielded by the data to a theoretical model consisting of 10 factors (groups of concepts) (Table S1). The statistical software used was SPSS, version 15 (SPSS Inc., Chicago, IL) for exploratory factor analysis and R statistical software, version 3.6.3 (Foundation for Statistical Computing, Vienna, Austria) for confirmatory factor analysis.

Lastly, the 37 concepts were divided into ten factors: morphostructural basic concepts linked to morphology, structure, function and the relationships among them (MBC); tissue organization concepts related to the elemental components of tissues (TO); hierarchical body organization concepts related to the levels of organization in the human body (HBO); organ histofunctional organization concepts related to the stromal and parenchymal nature of tissue components (OHO); concepts related to the histogenesis and development of tissues (HD); tissue functional state concepts related to the general activity of tissues (TFS); tissue engineering concepts related to the generation of artificial tissues (TE);microscopic magnification concepts related to magnification with different instruments (MM); microscopic examination analysis concepts related to histological techniques (MEA); and concepts related to histological information arising from the two‐dimensional observation of microscopic structures (HIO). The concepts pertaining to each factor are shown in Table S1.

### Statistical analysis

Cronbach's alpha was used to assess test reliability. The results were analyzed to identify differences among the different study groups in the concepts identified as TC. Average values and standard deviations were calculated for each factor. Two‐way ANOVA was used to compare the results obtained for each factor among the three groups. Thereafter, post‐hoc analyses with the Tukey test were used to detect specific pairwise differences between dentistry and medicine, dentistry and pharmacy, and medicine and pharmacy groups. Statistical tests were done with SPSS statistical package, version 15 (SPSS Inc., Chicago, IL) and the significance level was set at *P* < 0.05 for all tests. Effect sizes of the differences were calculated as Cohen's *d* (Δ), and were categorized as small (0 ≤ Δ < 0.333), medium (0.333 ≤ Δ < 0.666) or large (0.666 ≤ Δ < 1) based on benchmarks suggested by Cohen (1988).

## RESULTS

**FIGURE 1:**
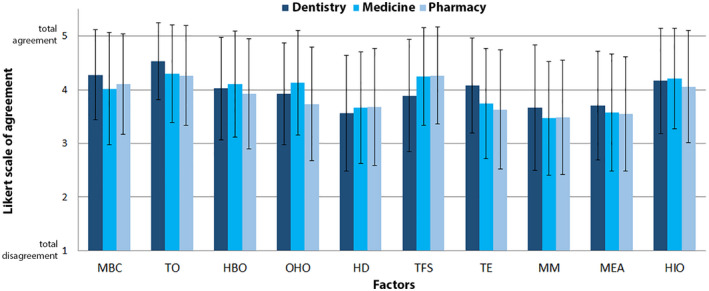
Overall scoring of threshold concepts by 64 dentistry students, 244 medicine students, and 102 pharmacy students (*n* = 410). The questionnaire was validated with exploratory factor analysis, which grouped 37 threshold concepts into ten different factors. Means (±SD) values for ten factors were obtained from the Likert scale scores ranging from 1 (total disagreement) to 5 (total agreement). HBO, hierarchical body organization; HD, histogenesis and development; HIO, histological information arising from two‐dimensional observation; MBC, morphostructural basic concepts; MEA, microscopic examination analysis; MM, microscopic magnification; OHO, organ histofunctional organization; TE, tissue engineering; TFS, tissue functional states; TO, tissue organization. The most relevant concepts for the learning of histology were those related to tissue organization, tissue functional states, and histological information arising from two‐dimensional observation. Tissue organization concepts received the highest scores from students in all degree programs

All participants in the study completed the HTCq, and scores were recorded for each TC and then clustered in 10 categories according to exploratory and confirmatory factorial analysis. The results for perceptions of each factor by dentistry, medicine and pharmacy students are shown in Table 2 and Figure 1. Individual scores for each concept are shown in Figure S2 to provide more detailed information. Cronbach's alpha resulted in 0.901 indicating acceptable test reliability.

**TABLE 2:** Scores of threshold concepts factors

Factor					D v M		D v Ph		M v Ph	
	Dentistry Mean (±SD)	Medicine Mean (±SD)	Pharmacy Mean (±SD)	Global Mean (±SD)	*p*‐value	Δ	*p*‐value	Δ	*p*‐value	Δ
Morphostructural concepts (MSC)	4.3 (±0.8)^a^, ^b^	4.0 (±1.0)	4.1 (±0.9)	4.1 (±1.0)	**0.001**	0.259	**0.026**	0.190	0.230	0.089
Tissue organization (TO)	4.5 (±0.7)^a^, ^b^	4.3 (±0.9)	4.3 (±0.9)	4.4 (±0.9)	**<0.001**	0.263	**<0.001**	0.316	0.708	0.044
Hierarchical body organization (HBO)	4.0 (±1.0)	4.1 (±1.0)	3.9 (±1.0)	4.0 (±1.0)	0.626	0.091	0.141	0.100	0.159	0.190
Organ histofunctional organization (OHO)	3.9 (1.0±)^b^	4.1 (±1.0)^c^	3.7 (±1.1)	3.9 (±1.0)	0.781	0.216	**0.018**	0.177	**0.007**	**0.388**
Histogenesis and development (HD)	3.6 (±1.1)	3.7 (±1.0)	3.7 (±1.1)	3.6 (±1.1)	0.475	0.106	0.612	0.110	0.116	0.010
Tissue functional states (TFS)	3.9 (±1.1)	4.3 (±0.9)	4.3 (±0.9)	4.1 (±0.9)	0.239	**0.383**	0.117	**0.383**	0.351	0.011
Tissue engineering (TE)	4.1 (±0.9)^a^, ^b^	3.7 (±1.0)^c^	3.6 (±1.1)	3.8 (±1.0)	**0.003**	0.339	**<0.001**	**0.436**	**0.007**	0.104
Microscopical magnification (MM)	3.67 (±1.2)	3.5 (±1.0)	3.5 (±1.1)	3.5 (±1.1)	0.107	0.185	0.474	0.162	0.338	0.019
Microscopic examination analysis (MEA)	3.7 (±1.0)	3.6 (±1.1)	3.6 (±1.1)	3.6 (±1.1)	0.111	0.112	0.087	0.144	0.660	0.028
Histological information arising from bidimensional observation (HIO)	4.2 (±1.0)	4.2 (±0.9) ^c^	4.1 (±1.1)	4.1 (±1.0)	0.455	0.053	0.062	0.098	**0.001**	0.154

Note

Mean and standard deviation of students' scores for their perception of threshold concepts. Number of participants: dentistry (*n* = 64), medicine (*n* = 244) and pharmacy (*n* = 102); total number of students (*n* = 410). Overall results are also shown as the average scores across all students. Statistically significant differences were identified by the bold‐faced font. Effect sizes of the differences were estimated as Cohen's *d* (Δ), and categorized as small (0 ≤ Δ < 0.333), medium (0.333 ≤ Δ < 0.666), or large (0.666 ≤ Δ < 1) based on benchmarks suggested by Cohen (1988).

Abbreviations: D, Dentistry; M, Medicine; Ph, Pharmacy.

^a^ Difference between Medicine and Dentistry.

^b^ Difference between Dentistry and Pharmacy.

^c^ Difference between Medicine and Pharmacy.

The overall results for students in all three groups showed that the most relevant concepts for learning histology were those related to tissue organization (4.4 ± 0.9),tissue functional states (4.1 ± 0.9), and histological information arising from two‐dimensional observation (4.1 ± 1.0). In fact, tissue organization concepts were perceived most clearly as TC by students in all groups.

However, analysis of the responses by students in different health science degree programs disclosed some differences among groups. Specifically, dentistry students highlighted the importance of morphostructural concepts (4.3 ± 0.8), whereas medical students considered that notions related to histological information arising from two‐dimensional observation (4.2 ± 0.9) and tissue functional states (4.3 ± 0.9) were more relevant. Pharmacy students emphasized the value of tissue functional states concepts (4.3 ± 0.9) for their learning of histology.

Dentistry students assigned significantly higher scores to morphostructural and tissue organization concepts than medical or pharmacy students (Figure 1). Morphostructural concepts were highly valued (mean score of 4.3 ± 0.8) by dentistry students, whereas medical student perceptions yielded a slightly lower mean score of 4.0 ± 1.0 (*P* = 0.001) and pharmacy students' mean score for these concepts was 4.1 ± 0.9 (*P* = 0.026). Similarly, the results for tissue organization concepts yielded a mean score of 4.5 ± 0.7 among dentistry students, with significantly lower scores among medical (4.3 ± 0.9; *P* < 0.001) and pharmacy students (4.3 ± 0.9; *P* < 0.001). There were also statistically significant differences among groups for organ histofunctional organization concepts. Although the difference between medical (4.1 ± 1.0) and dentistry students (3.9 ± 1.0) was not significant (*P* = 0.216), pharmacy students perceived these concepts to be of less relevance (3.7 ± 1.1), and their mean score was significantly lower than in the medicine (*P* = 0.007) and dentistry (*P* = 0.018) groups. Consequently, the effect size for the difference between medical and pharmacy students in their perception of organ histofunctional organization concepts was considered medium (Δ = 0.388).

Concepts related to histogenesis and development, hierarchical body organization, and tissue functional states were scored similarly by students in all three groups, with no significant differences (Figure S3). However, the effect size for differences in the perceived relevance of concepts related to tissue functional states was considered medium in dental students compared to medical (Δ = 0.383) and pharmacy students (Δ = 0.383).

Perceptions of tissue engineering showed remarkable differences among groups (Figure S3). The highest mean score was seen in dentistry students (4.1 ± 0.9), and this score was significantly higher than in medical (3.7 ± 1.0, *P* < 0.001) and pharmacy (3.6 ± 1.1, *P* = 0.007) students. In fact, the effect size for the difference in the perception of tissue engineering concepts was greatest between dentistry and pharmacy students (Δ = 0.436).

Concepts related to histological information arising from two‐dimensional observation of microscopic structures received a higher score from medical students (4.2 ± 0.9) compared to pharmacy students (4.1 ± 1.1; *P* = 0.001). Among the three groups, pharmacy students gave the lowest scores to microscopic examination analysis (3.6 ± 1.1) and magnification concepts (3.5 ± 1.1), although the differences among groups were not statistically significant (Figure S3).

## DISCUSSION

Recent decades have seen the appearance of several pedagogical theories characterized by the central positioning of the student in the learning process (Walder, 2014). One such theory, the Threshold Concepts learning framework, aims to identify the concepts most relevant for learning in a given discipline. Currently, newer studies have attempted to identify TC in a wide variety of disciplines such as nursing (McKillop et al., 2014), palliative medicine (O’Callaghan et al., 2020), economics (Randall et al., 2018), or physics (Serbanescu, 2017), among others.

As noted by Meyer and Land (2003), TC theory is grounded in the notion of pedagogy as a space of uncertainty (Shulman, 2005), and a specific concept can be defined as a TC if it is transformative, irreversible, integrative, and troublesome (Meyer & Land, 2005).

### Threshold concepts in health sciences

Different strategies have been proposed within health sciences curricula such as audio diaries (Neve et al., 2017), recording and viewing video discussions in the laboratory (Carstensen & Bernhard, 2008), or discussions about significant learning experiences during clinical sessions in general medicine (Vaughan, 2016) and pediatrics (Randall et al., 2018). Moreover, a TC‐based pedagogical framework has also been used to elucidate students' perceptions of medical professionalism (Neve et al., 2017), as well as central notions in psychology and bioethics (Collett et al., 2017). Of note, most of these approaches in medical education have focused on the clinical level, whereas basic and transversal subjects have received less attention. In this connection, Loertscher et al. described a process of TC identification in biochemistry that involved faculty members and graduate students, and resulted in the identification of four notions considered central for adequate progress in biomedical studies: steady state, biochemical pathway dynamics and regulation, the physical basis of interactions, and thermodynamics of macromolecular structure formation (Loertscher et al., 2014). However, the present study is the first of its kind to use the TC pedagogical framework to identify TC in histology. The histology TC identified in the present study are essential for medical education, as they constitute a pillar for further learning and comprehension of human pathology in medical and surgical curricula.

This study evaluates students' perceptions for the identification of TC in histology in the undergraduate degree programs in dentistry, medicine and pharmacy at the University of Granada (Spain). The study of histology in these curricula is intended to enable students to identify the tissue disorders or drug effects underlying human diseases (Pawlina & Ross, 2020); consequently, the identification of TC in histology holds the potential to considerably improve curricular design in health sciences in higher medical education. Further, after appropriate characterization of forgetting curves (which tend to decay with time), it would be of interest to follow up on these students when they reach the final year in their degree programs. This could lead to a more accurate picture of the TC identified in the present study.

Survey‐based studies can be a useful tool to characterize profiles associated with the perception of knowledge in conceptual, procedural and attitudinal dimensions (McKee et al., 2015; Tsu et al., 2018; Sola et al., 2019). In this connection, although the use of focus groups of practitioners (Tanner, 2011) or reflective writing pieces (Fouberg, 2013) can contribute to the identification of TC, a questionnaire‐based methodology was used in the present study for several reasons. First, a collaborative strategy combining academics' experience and students' perceptions of learning can offer a more inclusive model to identify the characteristics associated with TC, given that different actors are consulted at different times during the learning process. On one hand, descriptors such as “troublesome” and “transformative” may be better identified by students, i.e., the actors who are confronted with certain concepts for the first time during their university education; this experience may thus prompt them to analyze new concepts during their training. Narrative and semi‐structured interviews are considered an adequate method for assessing troublesomeness component of TC as they allow students to present their particular stories (Martindale, 2015). Nevertheless, these methods could lead to some bias as troublesomeness is not only indicative of TC, but also due to a lack of effort or motivation (Santisteban‐Espejo et al., 2020).

On the other hand, the integrative and irreversible nature of a TC may be better assessed through collaboration with academics, given that the experience of confronting different disciplines over the course of longer periods equips them to develop a more realistic, comprehensive view of these attributes. Survey‐based strategies combine the perspectives of both actors involved in the learning experience: academics propose certain concepts, which are then evaluated by students in terms of liminality, irreversibility, transformativeness, and their integrative nature. In fact, it has been recently reported the use of surveys designed by relevant experts as a method to identify TC in higher education (Kilgour et al., 2019).

### Threshold concepts in histology

Undoubtedly, the task of finding TC for a specific discipline is challenging, and as yet there is no gold‐standard technique to achieve this goal. However, collaboration between students and teachers appears to be a suitable empirical approach that can facilitate and optimize TC identification. In this connection, Cousin proposed “transactional curriculum inquiry”—a process of coordinated crosstalk among students, academics and education developers—as an effective partnership for the identification of TC in higher education (Cousin, 2009).

An important consideration in this regard is that the tool developed and described here should be constructed appropriately and validated with multivariate data analysis techniques such as exploratory factor analysis (Lucas da Rocha Cunha et al., 2018). Exploratory factor analysis clusters survey items with the strongest mutual correlations based on students' responses (Hair et al., 1995). The questionnaire used in the present study was validated with exploratory factor analysis, which grouped 37 threshold concepts into 10 different factors. In addition, the survey structure was further validated with confirmatory factor analysis, which indicated an acceptable goodness of fit.

Overall, students in dentistry, medicine and pharmacy identified concepts related to tissue organization, tissue functional states, and microscopic identification as the most relevant TC. It is important to highlight that students from different degree programs perceived the learning of histology as a process that requires integrating two dimensions: static concepts related to the constituent elements of tissues, e.g., the concept of cell or extracellular matrix, and dynamic concepts such as stem cells as tissue renewal substrates, and the euplasic, proplasic and retroplasic state of tissues. The complexity of integrating static and dynamic concepts may pose a considerable barrier for the comprehension of histology.

The findings of the present study disclosed some differences in students' perceptions among the three health sciences degree programs—a result that did not occur in isolation. For example, findings reported previously by Campos‐Sanchez et al. were consistent with the present results. Significant differences were reported in the motivational profiles of students from different health sciences degree programs (Campos‐Sanchez et al., 2014a), and related work found that the conceptions of learning also differed significantly between students in health sciences and non‐health sciences master's degree programs (Campos et al., 2018). In addition, research in veterinary sciences compared digital slides and traditional microscopy in the teaching of histological concepts (Mills et al., 2007; Brown et al., 2016), and several studies have implemented the TC framework to evaluate teaching experiences developed for veterinary students in the clinical setting (Lygo‐Baker et al., 2015; Alpi & Hoggan, 2016). In the present study, veterinary students' perceptions regarding histological TC could not be investigated because the university where this study was carried out does not offer a degree program in this discipline.

Dentistry students in particular identified concepts related to tissue organization and morphostructural concepts as the most relevant to them. In dentistry, morphological and functional concepts are closely interrelated, and this could hinder students' efforts to distinguish between them (Nanci, 2018). Nevertheless, in medicine, morphology, i.e., the study of the size, shape and the constituent parts of tissues, is well delimited from the physiological study of different tissue functions (Anyanwu et al., 2012; Sherer et al., 2014). Once identified, this conceptual divergence could serve to design pedagogical programs built on collaboration between students from different health sciences degree programs, and to enhance the development of interprofessional competencies in histology, as proposed in a previous study (Haber et al., 2021).

Medical students, for their part, perceived the learning of histological concepts associated with the two‐dimensional identification of microscopic structures as an area of concern. This attitudinal perception raises an important issue, given that microscopic identification skills are necessary not only for histology learning, but also for the learning of applied medical subjects such as human pathology (Braun & Kearns, 2008). In this connection, a recent systematic review contrasted the role of digital microscopy compared to conventional light microscopy in the learning of pathology (Rodrigues‐Fernandes et al., 2020). Learning to identify tissues correctly under a microscope supports solidifying principles and concepts, and adds a real‐life knowledge component that cannot be acquired through theoretical teaching only. The comprehension of two‐dimensional features of microscopic images will clearly be useful in students' subsequent learning in the health sciences curriculum, and may help them to attain better skills and competencies in clinical diagnosis in the future. In fact, modern histology, which is directly oriented to the resolution of clinical problems through the use of teaching methods such as the examination of histopathological slides (Chapman et al., 2020; Hoda & Hoda, 2021), is one of the pillars of the clinical and professional qualifications of future doctors.

Turning to another TC, medicine and dentistry students' perceptions of tissue engineering concepts (native tissues, artificial tissues, and cell, tissue and organ culture) differed significantly in comparison to pharmacy students. In medicine and dentistry degree programs, histology is conceived not only as a diagnostic tool, but also as a discipline oriented to the treatment of human diseases with artificial tissues as substitutes. This educational goal is especially relevant at present, because tissue engineering currently constitutes a consolidated area with a well‐defined cognitive framework which can be implemented in educational programs in association with histology (Saavedra‐Casado et al., 2017; Santisteban‐Espejo et al., 2018, 2019; Martin‐Piedra et al., 2020).

Different bioartificial tissues have been developed to functionally replace damaged bone (McDermott et al., 2019), cornea (Gonzalez‐Andrades et al., 2017; Rico‐Sanchez et al., 2019), peripheral nerve (Carriel et al., 2017; Huang et al., 2020), skin (Egea‐Guerrero et al., 2019), blood vessels (Chandra & Atala, 2019), cartilage (Park et al., 2020), and oral mucosa (Blanco‐Elices et al., 2020), among other tissues. Now that new biomimetic artificial tissues are available to treat previously untreatable conditions, tissue engineering should be taught not only as a new horizon in histological science, but also as an important element of educational programs in health sciences curricula (Griffith et al., 2006; Wyles et al., 2019).

Currently, therapeutic procedures inspired by tissue engineering methods such as guided tissue regeneration are widely used in dentistry for reconstruction after dental and periodontal lesions have been removed (Lang & Lindhe, 2015; Garzon et al., 2018; Bueno et al., 2020; Xu et al., 2020; Azim et al., 2021). It is likely that because of this clinical impact, medicine and dentistry students perceived tissue engineering concepts as more valuable and applicable to daily clinical practice, whereas pharmacy students did not share this perception.

For pharmacy students who participated in the present study, histology is not as important, as a transversal subject in their higher education program, as other subjects such as biochemistry and drug development, which are given more attention. Not surprisingly, histological concepts associated with tissue organization and tissue functional states were the TC valued most by pharmacy students. Histology, as taught in the pharmacy curriculum, is oriented toward knowledge of the histological structures functionally linked to drug metabolism and the absorption, distribution, and elimination of drug components (Pang et al., 2020). Consequently the comprehension of histological structures in organs such as the liver and kidneys is an important issue, because this knowledge is needed to further understand drug metabolism along with the therapeutic and possible side effects of drug compounds (Kleiner, 2018; Al‐Naimi et al., 2019).

In summary, this questionnaire‐based study provides evidence of an approach that can be used to identify different pedagogical profiles related to the teaching of histology in dentistry, medicine and pharmacy. The three groups of students who participated in the present study generally perceived the learning of microscopic structures of organs as a process that requires the harmonization of static and dynamic concepts.

### Limitations of the study

One of the limitations of this study is the use of a questionnaire developed originally in the Spanish language. In order to preserve the significance of the concepts defined in the questionnaire, versions in other languages should be tested only after accurate, validated translation. Another limitation is the fact that histology faculty members, rather than the students themselves, suggested the TC that were scored by students in the questionnaire. This approach has been criticized by some authors because many academics have learned these concepts some time ago, and may consequently not be in an ideal position to identify troublesomeness during the learning process, which is one of the essential characteristics of TC (Randall et al., 2018). Moreover, almost by definition, the different actors involved in the learning process (educators and students) are located on different sides of the liminal space. This fact may introduce disparities in how students and academics understand genuine TC. Consequently, the authors of the present study cannot state categorically that the faculty members involved in this study identified all potential concepts that might later be confirmed as TC by the students. To overcome this potential shortcoming, the reliability of the data could be further verified through discussion groups with students, who could confirm some of their questionnaire responses soon after analysis of the results.

## CONCLUSIONS

In conclusion, the results of this study are potentially useful to optimize the design of health sciences curricula. The identification of threshold concepts through students' perceptions appears to be a useful approach to improving the teaching and learning process in dentistry, medicine and pharmacy undergraduate curricula.

## CONFLICT OF INTEREST

The authors have no conflict of interest to declare.

## Supporting information

Fig S1

Fig S2

Fig S3

Table S1
