# Supplementary material for: Identification of histological threshold concepts in health sciences curricula: Students' perception
Source: Anat Sci Educ. 2022 Feb 8;16(1):171–82. doi: 10.1002/ase.2171 (PMC10078720; doi:10.1002/ase.2171)
Supplement: Supplementary file 1 — Fig S1 [file ASE-16-171-s002.docx]

**MALE  FEMALE  Age ____**

**How did you gain access to the University?:**

**University Entrance Exam - Calification____**

**Other ways - Please specify which___________________________**

**HISTOLOGICAL THRESHOLD CONCEPTS QUESTIONNAIRE (HTCq)**

*Threshold concepts* are defined as those concepts or knowledge that, for a given discipline, present the following features:

1. They should be **transformative**, i.e. once understood, the student's perception and understanding of the discipline should change radically.

2. They should be **irreversible**, i.e. once well understood, students are unlikely to forget them.

3. They should be **integrative**, i.e. once well understood, they should connect and build bridges within and across disciplines.

4. They should be generally **troublesome** for students to understand.

5. They should be generally **bounded** for a discipline.

**Please rate from 1 to 5 your level of agreement or disagreement that each of the following concepts can be considered a threshold concept for the learning of histology:**

**A- MORPHOSTRUCTURAL BASIC CONCEPTS**

**The concept of morphology**

*(Macroscopic and microscopic spatial configuration of a living organism or inert material and of the different units of which it is composed)*

Total 1 2 3 4 5 Total

disagreement      agreement

**The concept of structure**

*(A set of elements and the relationships that link them together without it being possible to characterize or define the elements independently of their relationships)*

Total 1 2 3 4 5 Total

disagreement      agreement

**The concept of microscopic structure**

*(Structure made up of microscopic elements and the relationships among them)*

Total 1 2 3 4 5 Total

disagreement      agreement

**The concept of the form-structure-function relationship**

*(Correlation between the morphological and structural characteristics of a microscopic entity and its functional activity)*

Total 1 2 3 4 5 Total

disagreement      agreement

**B- TISSUE ORGANIZATION CONCEPTS**

**The concept of cell**

*(Structural and functional unit of living organisms)*

Total 1 2 3 4 5 Total

disagreement      agreement

**The concept of cell population**

*(A group of cells of the same lineage or functional activity)*

Total 1 2 3 4 5 Total

disagreement      agreement

**The concept of tissue or associated cell population**

*(Supracellular level of organization formed by cells associated by juxtaposition or intercellular substances with a specific functional activity)*

Total 1 2 3 4 5 Total

disagreement      agreement

**The concept of dispersed cell population**

*(Supracellular level of organization made up of dispersed cells with a specific functional activity)*

Total 1 2 3 4 5 Total

disagreement      agreement

**The concept of extracellular matrix**

*(A set of fibrous and soluble molecules located in the intercellular space between the cells that form a tissue)*

Total 1 2 3 4 5 Total

disagreement      agreement

**The concept of stem cell as the basis for tissue renewal**

*(Stem cells are undifferentiated cells present in some tissues, with the capacity to self-renew and regenerate to form differentiated cells of one or more lineages, making tissue renewal possible)*

Total 1 2 3 4 5 Total

disagreement      agreement

**C- HIERARCHICAL BODY ORGANIZATION CONCEPTS**

**The concept of structural levels of organization**

*(Structural relationships organized in a hierarchical way from the simplest to the most complex)*

Total 1 2 3 4 5 Total

disagreement      agreement

**The concept of system**

*(An organized set of elements related by nature, structure, purpose, etc.)*

Total 1 2 3 4 5 Total

disagreement      agreement

**The concept of body organ**

*(Anatomical unit of the body, with characteristic shape and position, formed by the association of two or more tissues that converge to carry out a functional activity)*

Total 1 2 3 4 5 Total

disagreement      agreement

**The concept of the body apparatus**

*(The set of organs that contribute to a certain function in the organism)*

Total 1 2 3 4 5 Total

disagreement      agreement

**The concept of the body system**

*(Structural and functional unit of the human body consisting of an apparatus of the organism and the components of other apparatus that are functionally related to it)*

Total 1 2 3 4 5 Total

disagreement      agreement

**D- ORGAN HISTOFUNCTIONAL ORGANIZATION CONCEPTS**

**The concept of parenchyma**

*(Specific tissue of an organ)*

Total 1 2 3 4 5 Total

disagreement      agreement

**The concept of stroma**

*(Supporting tissue of an organ)*

Total 1 2 3 4 5 Total

disagreement      agreement

**E- HISTOGENESIS AND DEVELOPMENT CONCEPTS**

**The concept of histogenetic origin of tissues**

*(Tissues originate from the progressive differentiation of the three layers of blastoderm in the embryo)*

Total 1 2 3 4 5 Total

disagreement      agreement

**The ontogenetic concept of microscopic structures**

*(Evolution of the microscopic structure from the fertilized egg to its adult form)*

Total 1 2 3 4 5 Total

disagreement      agreement

**The phylogenic or phylogenetic concept of microscopic structures**

*(Evolution of microscopic structure in the course of the evolution of species)*

Total 1 2 3 4 5 Total

disagreement      agreement

**F- TISSUE FUNCTIONAL STATES CONCEPTS**

**The concept of the euplasic state in microscopic structures**

*(Orthotypic state or state of health and its variations)*

Total 1 2 3 4 5 Total

disagreement      agreement

**The concept of the proplasic state in microscopic structures**

*(State of increased general activity: phenomena of renewal, regeneration and repair aimed at recovery of the healthy status)*

Total 1 2 3 4 5 Total

disagreement      agreement

**The concept of the retroplasic state in microscopic structures**

*(State of decreased general activity: degeneration and ageing phenomena leading to loss of the healthy status)*

Total 1 2 3 4 5 Total

disagreement      agreement

**The concept of the injury state in microscopic structures**

*(State of alteration of microscopic structures related to loss of the healthy status)*

Total 1 2 3 4 5 Total

disagreement      agreement

**G- TISSUE ENGINEERING CONCEPTS**

**The concept of native tissue**

*(Tissue existing in the human organism)*

Total 1 2 3 4 5 Total

disagreement      agreement

**The concept of artificial tissue**

*(Tissue engineered for therapeutic application)*

Total 1 2 3 4 5 Total

disagreement      agreement

**The concept of cell, tissue and organ culture**

*(Laboratory culture of cells, tissue slices or organs for cytological and histofunctional studies or for the generation of artificial tissues)*

Total 1 2 3 4 5 Total

disagreement      agreement

**H- MICROSCOPIC MAGNIFICATION CONCEPTS**

**The concept of magnification in magnifying instruments**

*(Magnifying power of a lens or other optical instrument, expressing the number of times the optical system makes an object appear closer or larger)*

Total 1 2 3 4 5 Total

disagreement      agreement

**The concept of resolving power and limit of resolution in different magnifying instruments**

*(The resolving power is the ability of any optical system to perceive detail)*

*(The limit of resolution is the smallest distance that must exist between two points before they can be perceived as separate entities)*

Total 1 2 3 4 5 Total

disagreement      agreement

**The concept of microscopic units of measurement**

*(System of measurements used for microscopic quantities)*

Total 1 2 3 4 5 Total

disagreement      agreement

**I- MICROSCOPIC EXAMINATION ANALYSIS CONCEPTS**

**The concept of histological technique**

*(Set of steps carried out in the laboratory in order to maintain and make dead tissue visible with magnifying instruments)*

Total 1 2 3 4 5 Total

disagreement      agreement

**The concept of spatial vision in microscopic images**

*(Ability to imagine and situate microscopic images in two-dimensional or three-dimensional space)*

Total 1 2 3 4 5 Total

disagreement      agreement

**The concept of equivalent image**

*(Histological image that always reproduces the structure that exists in nature)*

Total 1 2 3 4 5 Total

disagreement      agreement

**The concept of artefact**

*(Histological image that does not reproduce the structure existing in nature due to the effect of processing: artefacts due to fixation, staining, etc.)*

Total 1 2 3 4 5 Total

disagreement      agreement

**The concept of dynamic vision in microscopic imaging**

*(The histological image is the portrait of a set of biological processes whose characteristics can be identified by different histological methods: histochemical, immunohistochemical, autoradiographic, etc.).*

Total 1 2 3 4 5 Total

disagreement      agreement

**J- HISTOLOGICAL INFORMATION ARISING FROM TWO-DIMENSIONAL OBSERVATION CONCEPTS**

**The concept of section orientation in relation to microscopic structures**

*(Ability to relate microscopic structure to the direction of sectioning: transverse, oblique, or longitudinal)*

Total 1 2 3 4 5 Total

disagreement      agreement

**The concept of topographic localization of microscopic structures**

*(Set of terms that identify the location of a given microscopic structure: apical, basal, proximal, distal, etc.)*

Total 1 2 3 4 5 Total

disagreement      agreement
