# Supplementary figures and images for: Identification of histological threshold concepts in health sciences curricula: Students' perception
Source: Anat Sci Educ. 2022 Feb 8;16(1):171–82. doi: 10.1002/ase.2171 (PMC10078720; doi:10.1002/ase.2171)

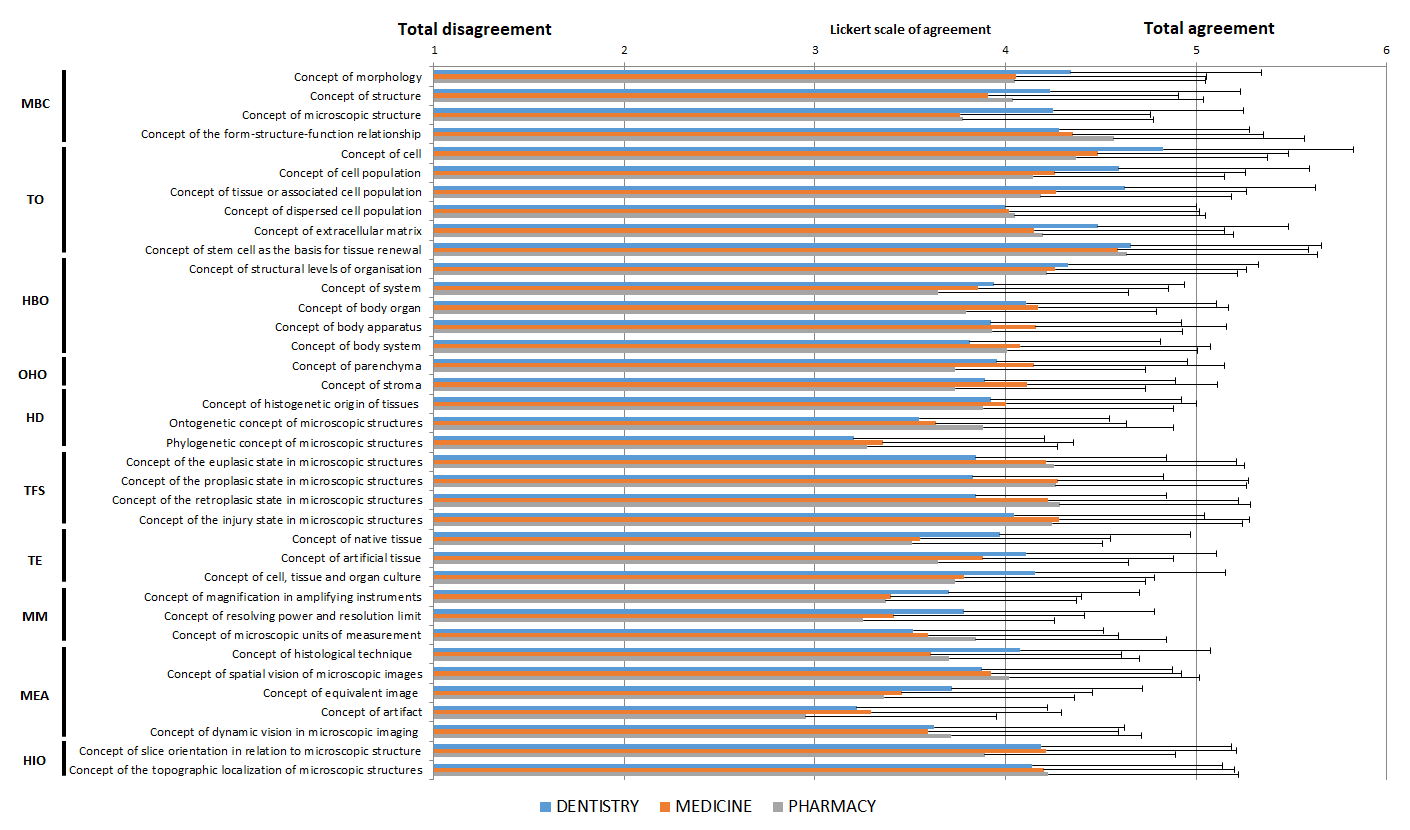

Supplement: Supplementary file 2 — Fig S2 [file ASE-16-171-s001.tif]

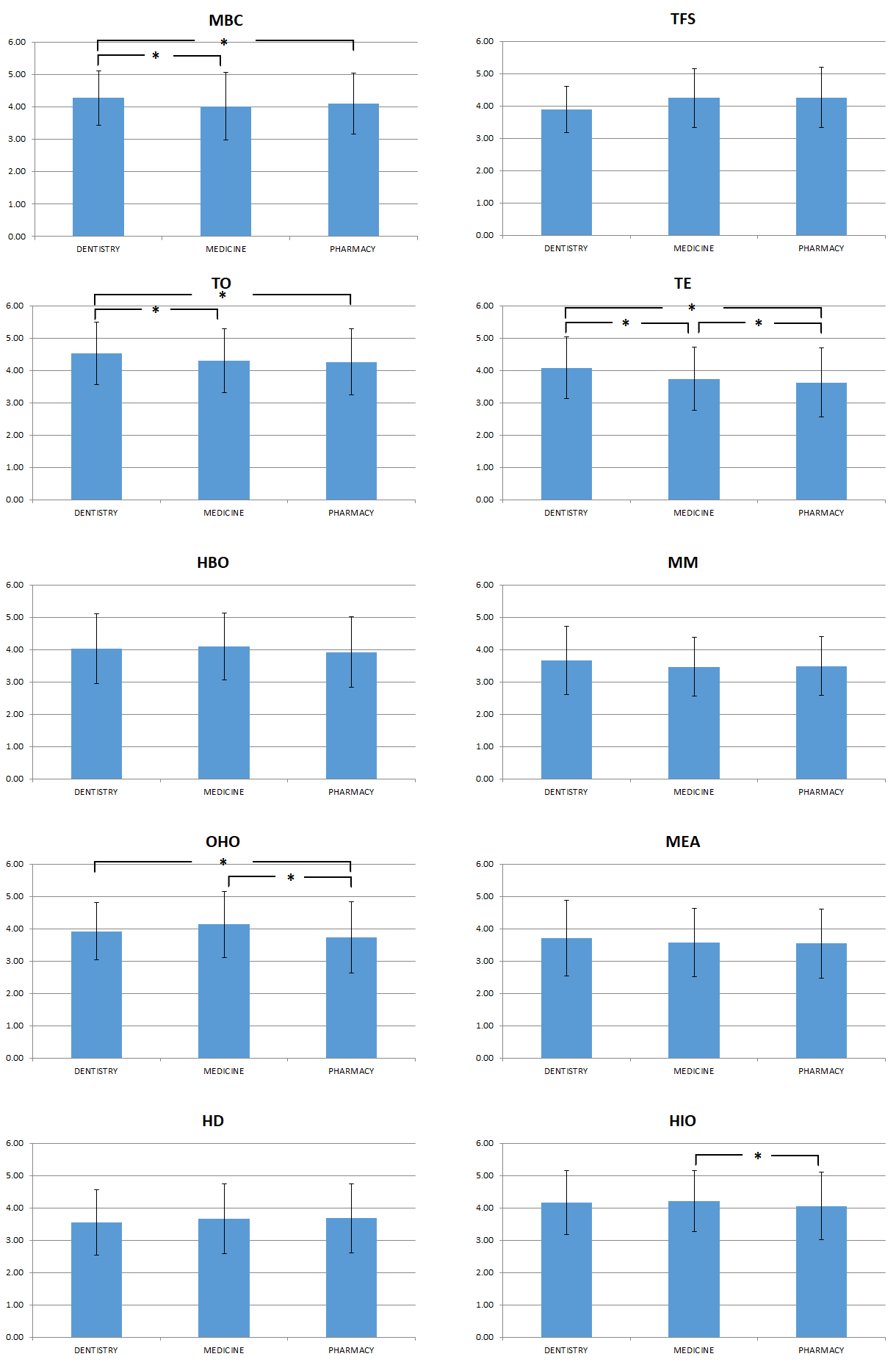

Supplement: Supplementary file 3 — Fig S3 [file ASE-16-171-s004.tif]
