# Supplementary material for: Identification of histological threshold concepts in health sciences curricula: Students' perception
Source: Anat Sci Educ. 2022 Feb 8;16(1):171–82. doi: 10.1002/ase.2171 (PMC10078720; doi:10.1002/ase.2171)
Supplement: Supplementary file 4 — Table S1 [file ASE-16-171-s003.docx]

| **Factor** | **Item** |
| --- | --- |
| **MORPHOSTRUCTURAL BASIC CONCEPTS (MBC)** | Morphology |
|  | Structure |
|  | Microscopic structure |
|  | Form-structure-function relationship |
| **TISSUE ORGANIZATION (TO)** | Cell |
|  | Cell population |
|  | Tissue or associated cell population |
|  | Dispersed cell population |
|  | Extracellular matrix |
|  | Stem cell as the basis for tissue renewal |
| **HIERARCHICAL**  **BODY ORGANIZATION (HBO)** | Structural levels of organisation |
|  | System |
|  | Body organ |
|  | Body apparatus |
|  | Body system |
| **ORGAN**  **HISTOFUNCTIONAL ORGANIZATION (OHO)** | Parenchyma |
|  | Stroma |
| **HISTOGENESIS AND DEVELOPMENT (HD)** | Histogenetic origin of tissues |
|  | Phylogenetic concept of microscopic structures |
|  | Ontogenetic concept of microscopic structures |
| **TISSUE FUNCTIONAL STATES (TFS)** | Euplasic state in microscopic structures |
|  | Proplasic state in microscopic structures |
|  | Retroplasic state in microscopic structures |
|  | Injury state in microscopic structures |
| **TISSUE ENGINEERING (TE)** | Native tissue |
|  | Artificial tissue |
|  | Cell, tissue and organ culture |
| **MICROSCOPICAL MAGNIFICATION (MM)** | Magnification in magnifying instruments |
|  | Resolving power and limit of resolution in different magnifying instruments |
|  | Microscopic units of measurement |
| **MICROSCOPIC EXAMINATION ANALYSIS (MEA)** | Histological technique |
|  | Spatial vision of microscopic images |
|  | Equivalent image |
|  | Artifact |
|  | Dynamic vision in microscopic imaging |
| **HISTOLOGICAL INFORMATION ARISING FROM**  **BIDIMENSIONAL OBSERVATION (HIO)** | Section orientation in relation to microscopic structures. |
|  | Topographic localization (apical, basal,…) of microscopic structures |
